# Injectable hybrid system for strontium local delivery promotes bone regeneration in a rat critical-sized defect model

**DOI:** 10.1038/s41598-017-04866-4

**Published:** 2017-07-11

**Authors:** Ana Henriques Lourenço, Nuno Neves, Cláudia Ribeiro-Machado, Susana R. Sousa, Meriem Lamghari, Cristina C. Barrias, Abel Trigo Cabral, Mário A. Barbosa, Cristina C. Ribeiro

**Affiliations:** 1 i3S - Instituto de Investigação e Inovação em Saúde, Universidade do Porto, Rua Alfredo Allen, 208, 4200 - 135 Porto, Portugal; 2 INEB - Instituto de Engenharia Biomédica, Universidade do Porto, Rua Alfredo Allen, 208, 4200 - 135 Porto, Portugal; 3 Faculdade de Engenharia, Universidade do Porto, Rua Dr. Roberto Frias, s/n, 4200-465 Porto, Portugal; 4 Faculdade de Medicina, Universidade do Porto, Serviço de Ortopedia, Alameda Prof. Hernâni Monteiro, 4200-319 Porto, Portugal; 5 ISEP – Instituto Superior de Engenharia do Porto, Instituto Politécnico do Porto, Rua Dr. António Bernardino de Almeida 431, 4249-015 Porto, Portugal; 6 ICBAS - Instituto de Ciências Biomédicas de Abel Salazar, Universidade do Porto, Rua de Jorge Viterbo Ferreira n. 228, 4050-313 Porto, Portugal

## Abstract

Strontium (Sr) has been described as having beneficial influence in bone strength and architecture. However, negative systemic effects have been reported on oral administration of Sr ranelate, leading to strict restrictions in clinical application. We hypothesized that local delivery of Sr improves osteogenesis without eliciting detrimental side effects. Therefore, the *in vivo* response to an injectable Sr-hybrid system composed of RGD-alginate hydrogel cross-linked *in situ* with Sr and reinforced with Sr-doped hydroxyapatite microspheres, was investigated. The system was injected in a critical-sized bone defect model and compared to a similar Sr-free material. Micro-CT results show a trend towards higher new bone formed in Sr-hybrid group and major histological differences were observed between groups. Higher cell invasion was detected at the center of the defect of Sr-hybrid group after 15 days with earlier bone formation. Higher material degradation with increase of collagen fibers and bone formation in the center of the defect after 60 days was observed as opposed to bone formation restricted to the periphery of the defect in the control. These histological findings support the evidence of an improved response with the Sr enriched material. Importantly, no alterations were observed in the Sr levels in systemic organs or serum.

## Introduction

The management of fractures and bone defects remains a significant challenge, and there is the need for improved therapeutic strategies^1^. Biological (autografts, allografts and xenografts) and synthetic bone grafts are currently used in clinical practice for bone repair. Because of their osteogenic potential and the absence of risks of immune rejection or disease transfer, autografts are clinically preferred. However, they are limited in supply, imply the additional morbidity of a harvest surgery and their properties and shape do not match exactly those of the bone to be replaced^2^. Intensive investigation is being carried out to produce synthetic bone grafts in order to overcome these problems. The use of injectable materials in bone regeneration, especially calcium phosphate based materials, presents several advantages, namely due to their adequate biological responses, osteoconductivity and mechanical properties^3–6^. These materials can be applied by minimally invasive surgical procedures, to efficiently fill-in cavities of non-uniform shapes, with no tissue damage and limited exposure to infectious agents, thus reducing patient discomfort and procedure-associated health costs. The addition of osteoinductive factors or osteoprogenitor/stem cells may improve bone repair, particularly in osteoporotic conditions, characterized by an impaired healing response^7–10^.

Oral administration of Strontium (Sr) ranelate has shown effectiveness in the prevention of both vertebral and non-vertebral osteoporotic fractures^11, 12^. Unlike other anti-osteoporotic agents widely used in clinical practice, such as bisphosphonates, estrogen, selective estrogen-receptor modulators (SERMs) and calcitonin, which inhibit bone resorption^9^, Sr ranelate also promotes bone formation^13–16^. Several *in vitro* studies show that Sr ranelate decreases bone resorption, by reducing osteoclast activity^13, 14, 17^, decreasing functional osteoclast markers expression^13^, disrupting osteoclasts cytoskeleton^14^, and increasing osteoclast apoptosis^18^. Simultaneously, it induces positive effects on osteoblastogenesis and osteoblast activity in different *in vitro* models^19^, namely by enhancing replication of preosteoblastic cells^14, 20–23^, increasing osteogenesis^14, 20, 24–26^, decreasing osteoblast apoptosis^21, 27^, and promoting terminal differentiation of osteoblasts into osteocytes^20^. Some pre-clinical studies performed in both normal and osteopenic/osteoporotic animal models confirmed these *in vitro* results, showing the beneficial effects of Sr ranelate on bone formation and remodeling^28–32^. Despite these important effects, cardiovascular safety of orally administered Sr ranelate has been questioned due to a small but significant increase in non-fatal myocardial infarctions^12, 33, 34^. Currently, there are strict indications and restrictions to its use^12^.

Nevertheless, Sr incorporation into biomaterials for bone regeneration may improve their regeneration potential. *In vivo* studies showed that doping calcium phosphate cements and other ceramics with Sr promotes bone repair^35–37^. A sustained delivery system for local release of Sr ions can obviate systemic complications with similar rates of bone formation at the site of implantation.

Current injectable bone defect methacrylate-based fillers have compression strengths much higher than that of cancellous bone, and the brittleness of calcium phosphate cements is a limitation^38, 39^. We have previously developed various types of injectable biomaterials for bone regeneration, namely calcium phosphate^40–42^ and calcium phosphate/alginate^43^ microspheres, as well as different types of bio-functional alginate hydrogels^44–47^. When combined, alginate can act as an appropriate vehicle for ceramic microspheres delivery and immobilization at the injury site. Alginate is a natural linear polysaccharide, biodegradable and biocompatible, extensively studied for biomedical applications^48, 49^. Although generally regarded as a bioinert material, since it does not elicit specific cell-matrix interactions, grafting of alginate with arginine-glycine-aspartic acid (RGD) peptides is an effective strategy to provide appropriate guidance signals to promote cell adhesion and facilitate cell colonization^50^. Alginate forms hydrogels under mild chemical conditions, in the presence of divalent cations, such as calcium (Ca) and Sr, through a cytocompatible physical gelation process. These cations bind homoguluronic blocks in adjacent alginate chains in a cooperative manner (egg-box model) producing a crosslinked hydrogel network^51, 52^.

Recently, we developed an injectable hybrid system that consists of ~500 µm diameter hydroxyapatite (HAp) microspheres doped with Sr, embedded in a functionalized alginate matrix crosslinked *in situ* with Sr^53^. As a vehicle for the microspheres delivery, functionalized alginate with RGD peptides was used, providing a scaffold for cell adhesion and migration and allowing for the injectability of the system. However, the use of hydrogels in bone tissue engineering is limited by low mechanical properties and the non-applicability in load-bearing conditions^54^. The packing of the microspheres upon delivery raises the compression strength of the material^53^, and alginate creates an interconnected 3D network adequate for the invasion of blood vessels and cells^55^. Moreover, the presence of Sr in both components of the system provides two different release routes upon degradation of the materials. This system presents a clinically relevant compromise between adequate injectability, gelation time and final compression strength^53^. Moreover, *in vitro* studies showed that this Sr-hybrid scaffold promotes sustained release of Sr^2+^, supports human mesenchymal stem cells adhesion, survival and osteogenic differentiation, and inhibits osteoclasts differentiation and activity, as compared to a similar Sr-free system^56^.

In the current study we aim to evaluate the *in vivo* response to the designed Sr-rich hybrid system and its influence on new bone formation using a rat metaphyseal femoral critical-sized defect model, compared to a similar Sr-free material. The proposed system is expected to provide an adequate therapeutic approach to fill-in bone defects by minimally invasive surgery, while acting as a scaffold for local Sr^2+^ release to promote bone regeneration.

## Results

### Radiographical analysis of bone and biomaterial

**Figure 1:**
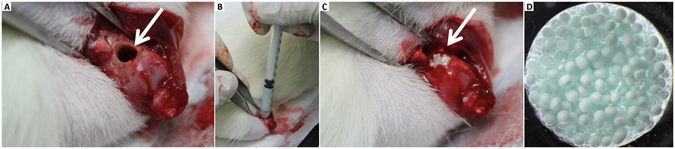
*In vivo* intraoperative setting. Critical sized defect created in the distal femur (**A**). Injection of the hybrid material using a 1 mL syringe (**B**) and filled defect (**C**). Detail of the hybrid system, composed of HAp microspheres embedded in a RGD-alginate hydrogel (**D**).

A cylindrical defect with 3 mm diameter and depth of approximately 4 mm was drilled in the lateral condyle of the right femur (Fig. 1A). The defect was filled with the biomaterial, injected using a 1 mL syringe (Fig. 1B,C). A picture of the Sr-hybrid material used is shown in Fig. 1D, where spherical particles (Sr-HAp microspheres) are embedded within a transparent hydrogel (Sr crosslinked RGD-alginate).

**Figure 2:**
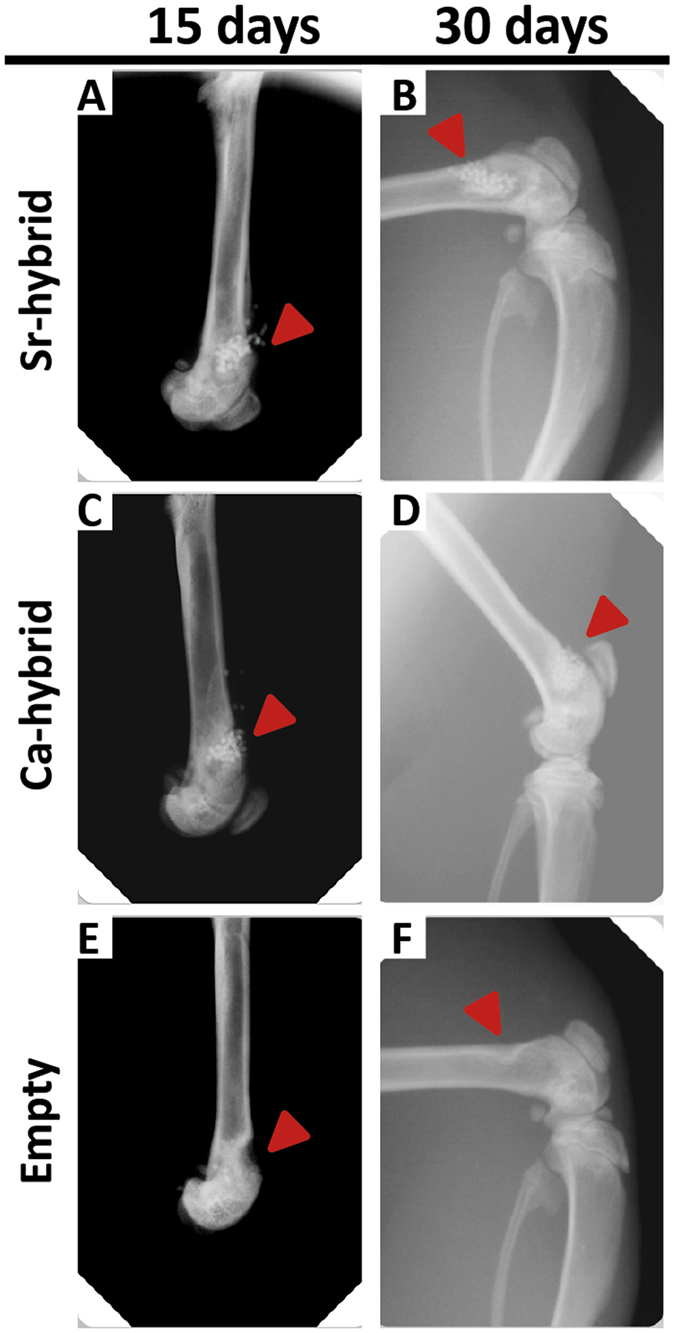
Radiographic imaging follow-up. Representative lateral X-Ray images from right rat femurs of Sr-hybrid (**A** and **B**), Ca-hybrid-filled (**C** and **D**) and empty (**E** and **F**) defects at 15 days (**A**,**C** and **E**) and 30 days (**B**,**D** and **F**) post-surgery. Arrows pinpoint site of created defect, filled or empty. Microspheres can be observed as circular objects more radiopaque than bone.

Rat femurs were imaged by X-ray along the experimental period allowing for a follow-up at 15 and 30 days. Representative images of defects filled with materials (Sr-hybrid and Ca-hybrid) or empty defects are shown in Fig. 2. In hybrid-filled defects (Fig. 2A to D), microspheres are located inside the created bone defect (arrows in the images), where the higher radiopacity of the HAp microspheres allowed for the easy monitoring using X-ray. Microspheres were homogeneously distributed within the defects (Fig. 2A to D) and were still detected at day 30, a non-invasive mid-term follow-up (Fig. 2B and D). Empty defects were also imaged (Fig. 2E and F) and the defect could still be observed after 30 days, confirming it to be of a critical size.

### Micro-CT morphometric 3D evaluation

**Figure 3:**
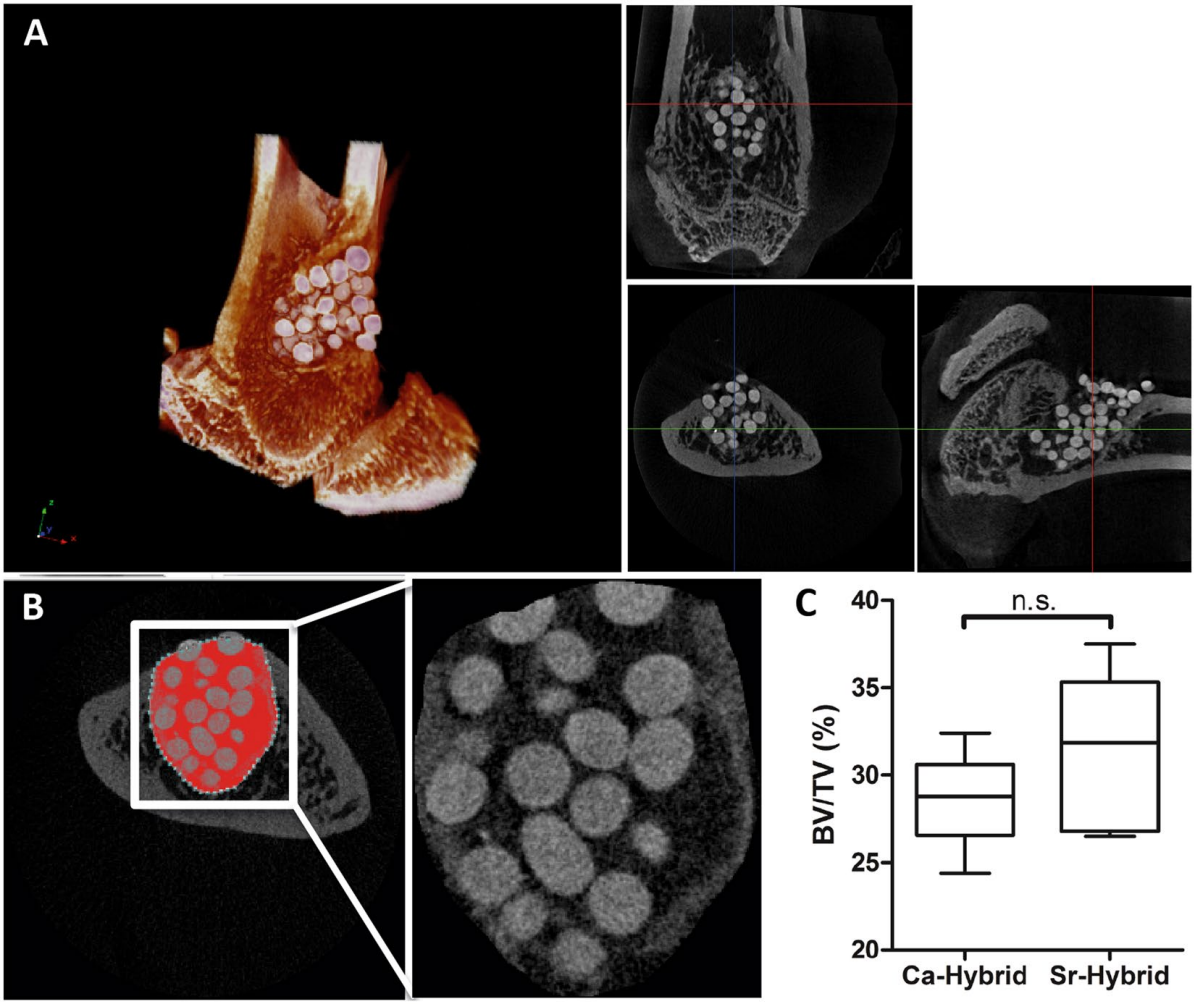
Micro-CT analysis of distal femur after 60 days of implantation. 3D reconstructed image and respective orthogonal slices of micro-CT acquisition of the femur with Sr-hybrid filled defect (**A**). Morphometric analysis approach used to quantify new bone formation: transversal micro-CT slice of a femur injected with Sr-hybrid after 60 days of implantation highlighting ROI (**B**). (**C**) New bone formed (BV/TV, %) after 60 days of implantation of Sr-hybrid and Ca-hybrid materials. Data presented as box-plot with median and min to max whiskers of n = 5 samples (n.s. – statistically non-significant).

Micro-CT analysis was performed 60 days post-implantation (Fig. 3) to evaluate new bone formation at the defect site and to assess the spatial distribution of ceramic microspheres within the lesion. In Sr-hybrid filled defects (Fig. 3A), microspheres were homogeneously distributed inside the defect with no apparent degradation, with preserved size and without modifications in shape. Similar results were found in Ca-hybrid filled defects. Furthermore, and particularly in Sr-hybrid samples, centripetal bone colonization could be observed by new bone formation surrounding the ceramic microspheres and with the development of new bone trabeculae in the periphery of the defect. 3D morphometric analysis was performed using five femurs per group, where the ROI was defined in binary images (Fig. 3B) and the percentage of new bone formed (bone volume fraction, BV/TV) was calculated. Values of (31.5 ± 1.7) % and (28.6 ± 1.1) % (BV/TV (%), mean ± SE) of new bone was measured in animals that received the Sr-hybrid and Ca-hybrid materials, respectively (Fig. 3C).

### Histological evaluation of bone/biomaterial interface

**Figure 4:**
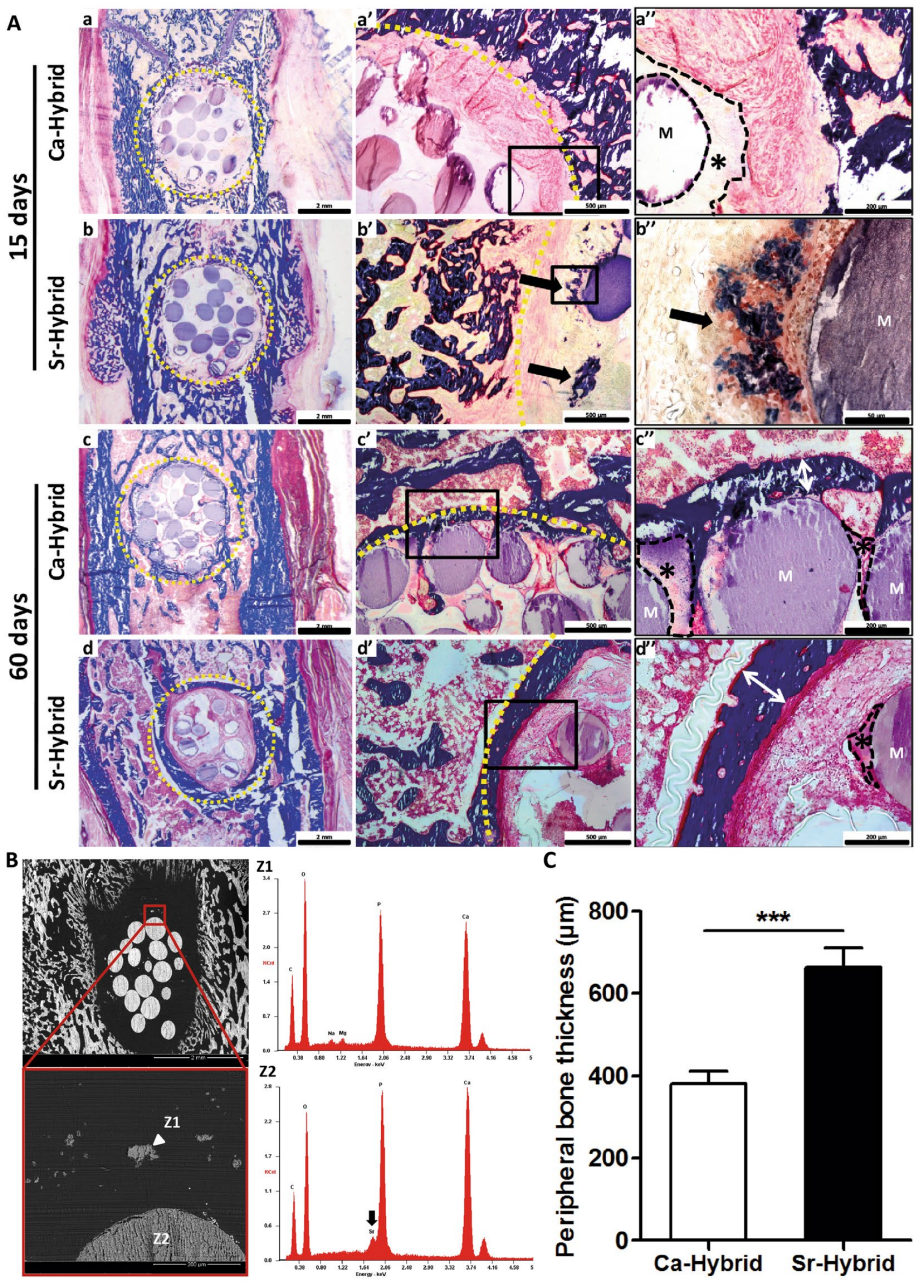
Histological evaluation of bone/biomaterial interface in femurs implanted with Sr-hybrid and Ca-hybrid systems. Coronal histological sections of critical sized defect created in the distal femur, 15 and 60 days post-implantation (**A**). Global view of the created defects filled with Ca-hybrid (a and c) and Sr-hybrid (b and d) materials, at 15 (a and b) or 60 days (c and d) post-implantation, stained with MT (a to d, dashed yellow line circling the created defect area). Interface bone/biomaterial, 15 days (a’ and b’) and 60 days (c’ and d’) post-implantation, with Ca-hybrid (a’ and c’) and Sr-hybrid (b’ and d’) systems, and higher magnification of rectangle (a” to d”, collagen/bone in blue, yellow dashed line – bone/biomaterial interface, * – alginate, M – microspheres, black arrows – new bone). (**B**) SEM image of histological section of Sr-hybrid filled defect 15 days post-implantation with EDS spectra of new bone (Z1) found near the microsphere and microsphere (Z2). (**C**) Thickness of peripheral bone found around the defect (as represented with arrows in **A** c” and **A** d”), 60 days post-implantation of Sr-hybrid and Ca-hybrid systems. Data presented as mean ± SEM of 20 different random locations of trabecular bone found around the defect of n = 5 animals/group, 3 sections/animal. Asterisks show statistically significant differences (***p < 0.001).

In Fig. 4A representative images of femurs with defects filled with Sr-hybrid or Ca-hybrid materials, at days 15 and 60, are portrayed. A global view of the defects and materials (Fig. 4Aa to d), as well as a more detailed view of the periphery of the defect (Fig. 4Aa’ to d’ and magnifications a” to d”), are given. Histological analysis at day 15 post-implantation showed that the created defects exhibited similar diameter and were filled with approximately 15 to 18 microspheres (Fig. 4Aa,b). As early as 15 days post-implantation, all animals showed, to some extent, newly formed bone at the periphery of the defect (Fig. 4Aa’,b’). Sr-hybrid implanted defects also showed new bone formation in close contact with the microspheres, distant from the periphery of native bone (Fig. 4Ab”, arrow). SEM images and EDS analysis of this newly formed bone in close vicinity of the microspheres is shown in Fig. 4B. The results confirmed the high content of calcium and phosphate, and a Ca/P ratio in accordance with normal bone composition (Z1 in Fig. 4B), and different from the elemental analysis of the microspheres (Z2 in Fig. 4B), where Sr was also identified.

After 60 days, new bone formation at the periphery was observed in both materials. Sr-hybrid implanted defects exhibited a thicker trabecular bone structure at the periphery of the defect (Fig. 4Ad’,d”), when compared to the Ca-hybrid group (Fig. 4Ac’,c”). The quantification of new bone formed at the periphery of the defect revealed a statistically significant thicker bone structure in Sr-hybrid group (662.4 ± 48 µm) in contrast to a thinner bone formation (381.1 ± 29 µm) in the Ca-hybrid group (mean ± SE, p < 0.001, Fig. 4C).

### Histological evaluation of the center of the defect

**Figure 5:**
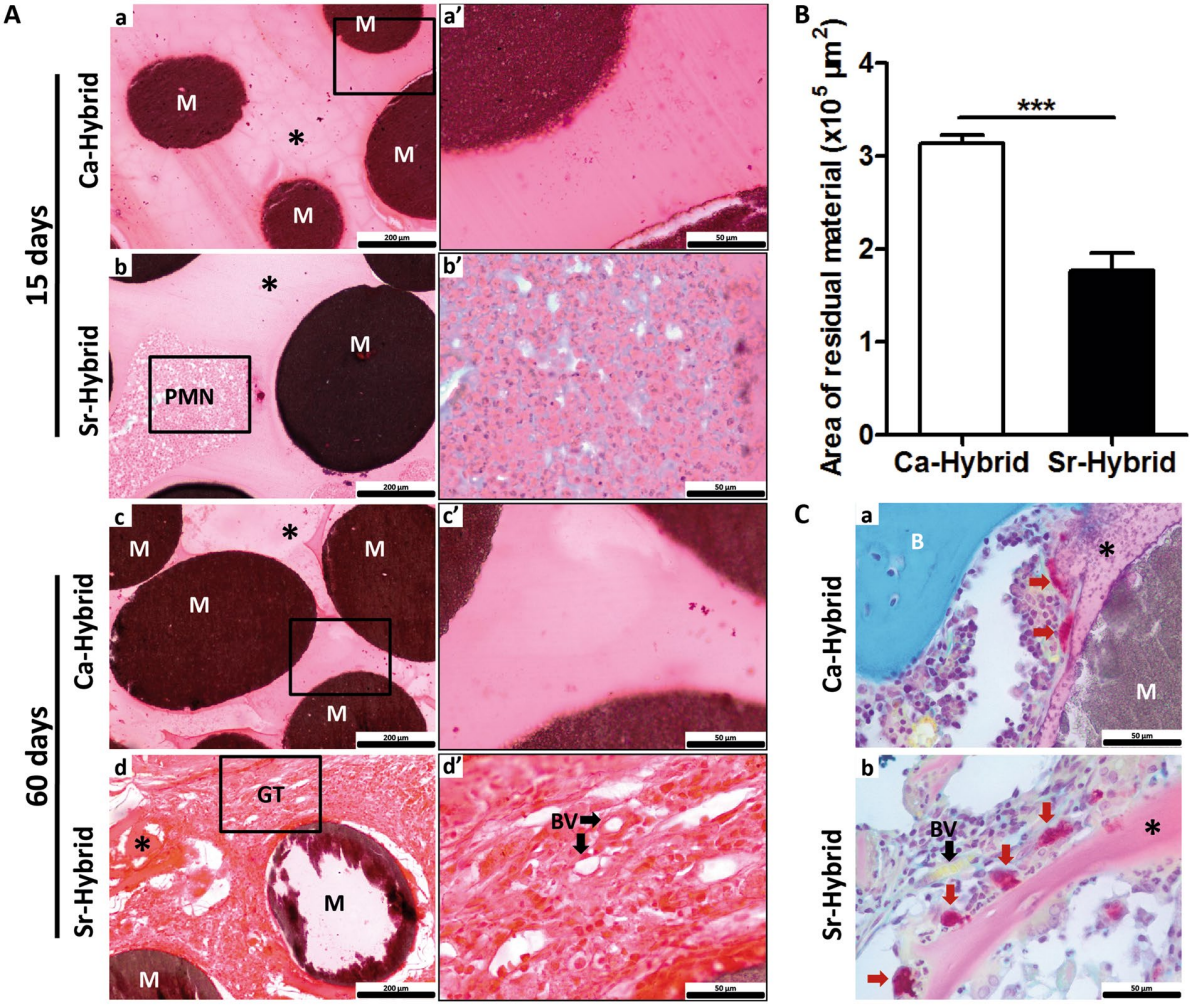
Histological analysis of the center of the defect in femurs implanted with Sr-hybrid and Ca-hybrid systems. Detailed view of the center of the defect implanted with Ca-hybrid (a and c) and Sr-hybrid (b and d) materials, stained with H&E, after 15 (a and b) and 60 days (c and d) of implantation, with higher magnification of rectangle (a’ to d’, **A**). (**B**) Area of residual material (microspheres and alginate) found within the defect area, 60 days post-implantation of Sr-hybrid and Ca-hybrid systems. Measurements were performed by delimiting the area of material in MT stained sections using ImageJ software, n = 5 animals/group, 3 sections/animal. Data presented as mean ± SE and asterisks show statistically significant differences (***p < 0.001). (**C**) TRAP-LG staining images of Ca-hybrid (a) and Sr-hybrid (b) systems filled defects, 60 days post-implantation. (M – microspheres, * – alginate, PMN – Polymorphonuclear neutrophils, GT – Granulation Tissue, BV – blood vessels, red arrows – Osteoclasts).

Representative images of the center of the defect in both groups are shown in Fig. 5A. Although no evident differences were observed in the diameter of the microspheres with time of implantation, alginate showed different behavior between groups. After 15 days, some alginate was observed surrounding microspheres in both experimental groups (Fig. 5Aa and b). In Sr-hybrid group, higher cell invasion at the center was observed, mainly of polymorphonuclear neutrophils (PMN) (Fig. 5Ab and b’).

**Figure 6:**
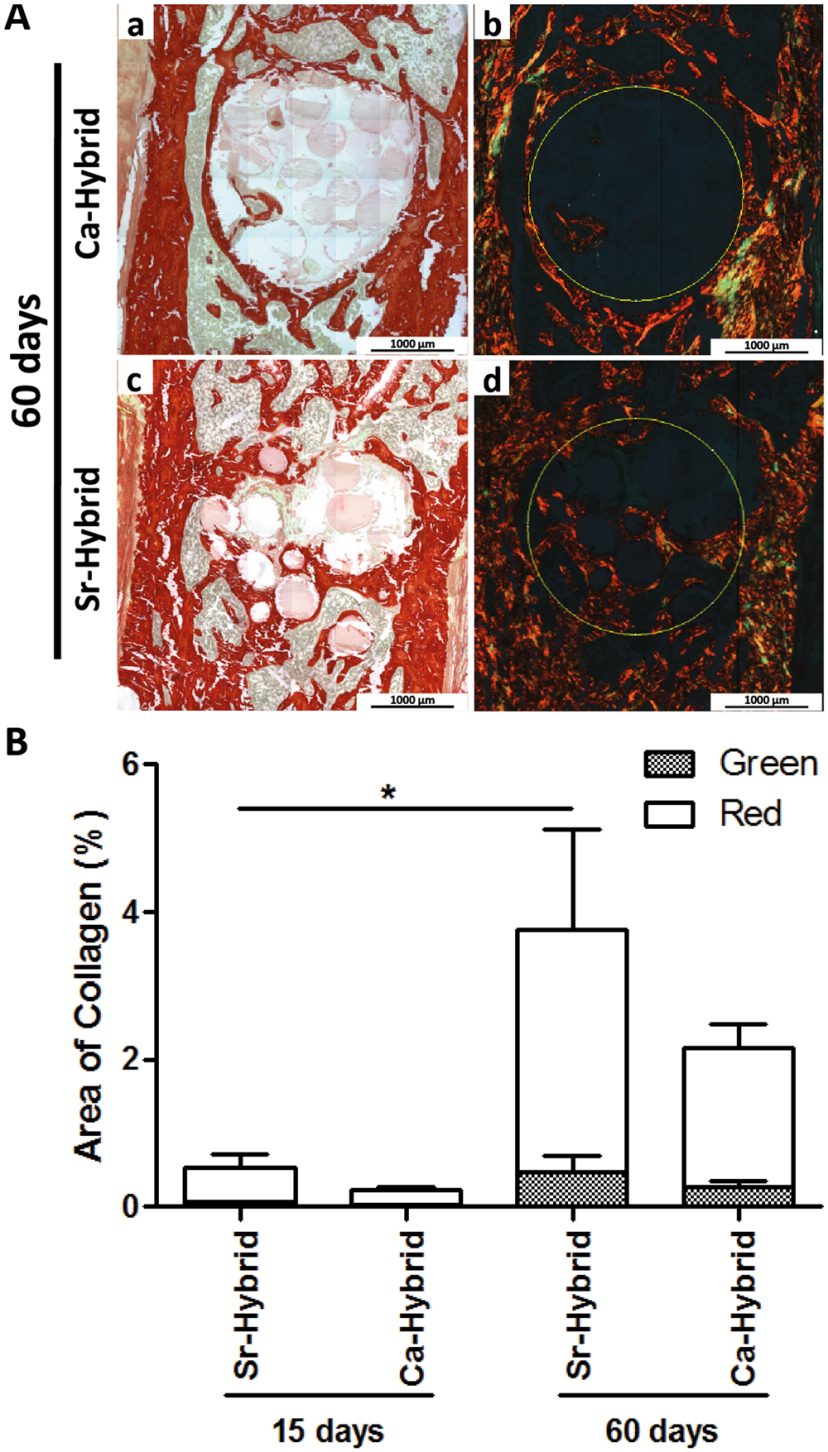
Quantification of birefringent collagen fibers by PSR-polarization method in the center of the defect. PSR staining visualized under conventional light (a and c) and polarized light (b and d) to identify different collagen fiber types, 60 days post-implantation of Ca-hybrid (a and b) and Sr-hybrid (c and d) systems (**A**). PSR stains collagen in red (conventional light) and collagen fibers are specifically birefringent in polarized light (green - thin fibers/type III; red - thick fibers/type I, MosaicX image, original magnification 20x). Fixed region of interest (ROI) used for quantification is demonstrated in b and d. (**B**) Quantification of area of green or red birefringent collagen fibers in the center of the defect in Sr-hybrid and Ca-hybrid filled defects, 15 and 60 days post-implantation. Data presented as mean ± SE of n = 5 animals/group, 3 images/animal. Asterisks show statistically significant differences (p < 0.05) between groups for red collagen data.

After 60 days, alginate was still present in both groups (Fig. 5Ac and d), although higher degradation was observed in Sr-hybrid filled defects. A statistically significant decrease in the area of residual material (alginate + microspheres) present on the defect site was observed in Sr-hybrid group with an area of 1.77 ± 0.2 × 10^5^ µm^2^ compared to 3.14 ± 0.1 × 10^5^ µm^2^ in Ca-hybrid group (mean ± SE, p < 0.001, Fig. 5B). In a detailed microscopic analysis of the center of the Sr-hybrid filled defects (Fig. 5Ad and d’) granulation tissue could be observed, with the presence of blood vessels (Fig. 5Ad’) and osteoclasts (Fig. 5Cb). In contrast, in Ca-hybrid group osteoclasts were found only at the periphery of the defect (Fig. 5Ca). Furthermore, PSR staining images under conventional light (Fig. 6Aa and c) showed the presence of collagen (in red) within the area of the defect in Sr-hybrid (Fig. 6Ac) at a higher extent than in Ca-hybrid filled defect after 60 days (Fig. 6Aa). The use of PSR-polarization method (Fig. 6Ab and d) allowed for the quantification of different types of collagen fibrils, i.e. green and red, which are associated with thin/immature/type III and thick/mature/type I collagen, respectively. The quantification was performed within the central area of the defect using a fixed ROI (diameter = 2.4 mm, yellow circle in Fig. 6Ab and d) and results are shown in Fig. 6B. As expected, an increase in the percentage of red/type I collagen was observed from day 15 to day 60 in both groups. However, 60 days post-implantation, a slightly higher percentage of red/type I collagen was measured in the central defect region in Sr-hybrid group ((3.3 ± 1.3) %, mean ± SE) compared to Ca-hybrid group ((1.9 ± 0.3) %, mean ± SE).

### Evaluation of Sr systemic effect

**Figure 7:**
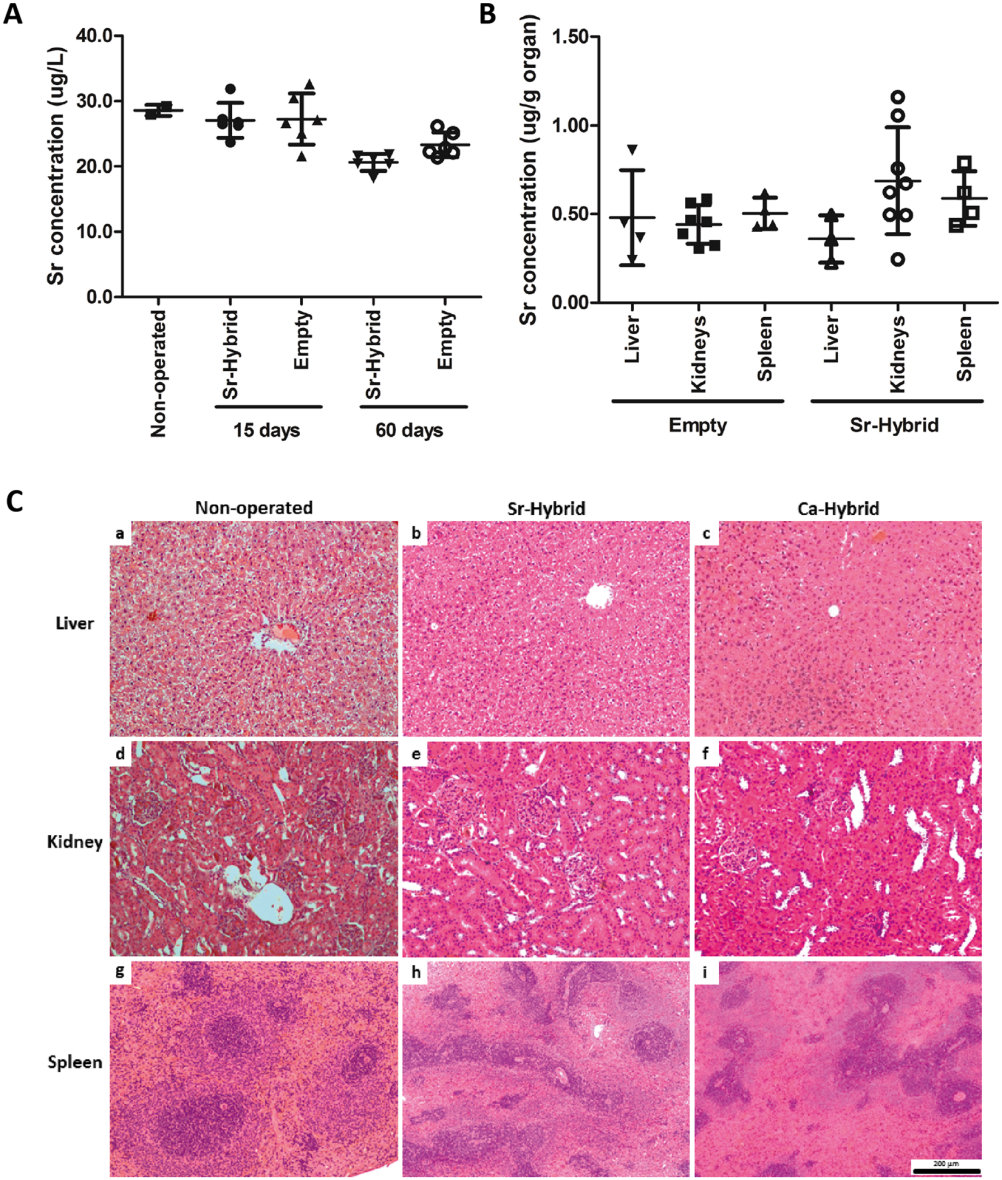
Strontium systemic evaluation. Sr concentration found in serum in non-operated, empty and Sr-hybrid implanted animals at days 15 and 60 post-surgery (**A**). Data presented as mean ± SD of 6 samples/group/time point and 2 samples non-operated. Sr concentration found in the liver, kidneys and spleen of animals implanted with Sr-hybrid and empty defect, 60 days post-surgery (**B**). Data presented as mean ± SD of 4 samples/group/time point. Representative histological sections from liver (a to c), kidney (d to f) and spleen (g to i) of non-operated (a, d and g), Sr-hybrid (b, e and h) and Ca-hybrid implanted (c, f, and i) animals, 60 days post-surgery (C).

Sr levels were quantified in serum (Fig. 7A) and organs (Fig. 7B) associated with excretory/filtration functions, such as liver, spleen and kidneys, by ICP-AES analysis, to evaluate the safety of the designed Sr-hybrid system. The Sr levels in serum of animals that were subjected to Sr-hybrid implantation (27.05 ± 2.7 µg/L after 15 days and 20.61 ± 1.3 µg/L after 60 days) were not statistically different from those in empty defect animals (27.26 ± 3.9 µg/L after 15 days and 23.31 ± 1.9 µg/L after 60 days) or non-operated animals (28.59 ± 0.8 µg/L, mean ± SD). Even after 60 days of implantation, no increase in Sr was found in serum (Limit of quantification, LOQ – 5 μg/L).

Sr quantification in organs at 60 days post-implantation supports results from measurements in serum. No statistical significant differences were observed between empty defect animals (0.44 ± 0.1 µg/g kidney, 0.50 ± 0.1 µg/g spleen, 0.48 ± 0.3 µg/g liver, mean ± SD) and Sr-hybrid group (0.69 ± 0.3 µg/g kidney, 0.59 ± 0.2 µg/g spleen, 0.36 ± 0.1 µg/g liver, mean ± SD). Moreover, histomorphological analyses were also performed in histological sections of organs after 60 days (Fig. 7C). In Sr-hybrid implanted group no morphological alterations at macro or microscopic level were observed, when compared to non-operated animals. Analysis of Ca-hybrid group presented similar results, with no alterations observed.

## Discussion

In this study the *in vivo* response to an injectable Sr-rich hybrid system, composed of Sr-doped HAp microspheres embedded in an Sr-crosslinked RGD-alginate hydrogel, as compared to a similar Sr-free system (Ca-hybrid material), using a rat metaphyseal femoral critical-sized defect model, is presented.

The designed hybrid system aims at providing adequate mechanical support in the early phases of bone formation and gradual replacement of the artificial scaffold by newly-formed bone with adequate function and mechanical properties. The use of hydrogels is a promising approach in skeletal regenerative medicine^49, 57–59^. Alginate has been used due to its biocompatibility, low toxicity, and mild gelation in the presence of divalent cations. Therefore alginate gels act as a natural extracellular matrix mimic which can be tuned to deliver bioactive agents and cells to the desired site, creating space for new tissue formation and control the structure and function of the engineered tissue^49^. Other works have incorporated alginate in self-setting cements, for improving injectability, cohesion and compression strength^60–62^. In the present study, the ability of alginate to form hydrogels *in situ* acting as a carrier for HAp microspheres under cytocompatible conditions, was explored. In agreement with our previous results^53^, the system showed to be adequate for minimally invasive implantation. A conventional syringe can be used to manually inject the material, perfectly filling complex defects and, once set, creating a 3D matrix with homogeneous distribution of microspheres. Furthermore, alginate was modified with RGD peptides to provide biological cues for promoting cell adhesion and colonization^50, 63^. The main disadvantage regarding load-bearing application is alginate low mechanical properties which can be overcome through the reinforcement with ceramic components, application in non load-bearing areas, and the concomitant use of fixation devices^54^. With an alginate-to-microspheres weight ratio of 0.35, and microspheres with average diameter of 530 µm, the hybrid system allows for a good compromise between mechanical resistance and adequate space between particles (approximately 220 μm), which is expected to facilitate *in situ* cell colonization and invasion by blood vessels^53, 55^.

Deficient bone healing is expected to occur, especially in osteoporotic conditions. The use of crystalline HAp in the microspheres, with low degradation rate, ensures its permanency at the injury site for longer periods post-implantation, therefore allowing for a mechanical reinforcement of the defect. In this work, the high radiopacity of microspheres allowed for an easy follow-up *in vivo* of the material using conventional radiological imaging. In clinical practice, this comes as highly advantageous since regular X-rays are required to assess bone healing.

In this system we used alginate crosslinked by internal gelation with Sr^2+^ as a vehicle for the Sr-doped HAp microspheres. Sr was incorporated both in the hydrogel and the microspheres, which present different release kinetic profiles resulting in sustained Sr^2+^ release for long periods of time (AH Lourenço *et al*., unpublished results). In other *in vivo* studies, Sr has been found to enhance bone formation^35, 37, 64, 65^. For example, Banerjee *et al*. studied the effect of doping β-TCP with MgO/SrO on bone formation in Sprague-Dawley rats^64^. Doped β-TCP promoted more osteogenesis and faster bone formation than pure β-TCP. In critical calvaria defects of an ovariectomized rat model, macroporous Sr-substituted scaffolds showed superior osteoinductive activity to enhance early bone formation, and could also stimulate angiogenesis compared with calcium silicate scaffolds^65^. The current study showed that Sr-hybrid system presents bioactive properties, promoting cell migration, implant vascularization and supports bone ingrowth. Newly formed bone developed in close contact with the material, without any fibrous interface, growing in a centripetal manner, in continuity with the surrounding host trabecular bone, indicates a good integration with the host tissue. Newly formed bone percentages is in agreement to those observed in other works testing similar materials^66, 67^ and a trend towards greater new bone formation was observed in Sr-hybrid filled defects.

Major histological differences were observed between the two groups. Higher cell invasion was seen at the center of the Sr-hybrid filled defects at 15 days post-implantation, with the presence of PMN. Bone injury elicits an inflammatory response that is beneficial to healing when acute and highly regulated. Inflammatory cells are recruited to the site of injury for clearance of pathogens and maintenance of bone homeostasis^68^. This higher cell invasion correlates with higher material degradation and bone tissue formation seen after 60 days. We may assume that Sr induces faster bone healing, possibly due to a faster resolution of inflammation, tissue repair and remodeling. This can be appealing since the presence of Sr may be modulating the inflammatory response, a current trend in bone regeneration strategies regarding the development of biomaterials^69–71^.

Earlier bone formation was identified in close contact with the microspheres in Sr-hybrid system, highlighting higher osteoinductivity when compared to Ca-hybrid system which was evidenced by the thicker trabecular bone structure at the periphery of the defect observed in Sr-hybrid group 60 days post-implantation. Cardemill *et al*. have also found major differences in the topological distribution of the formed bone in association with Sr-doped calcium phosphate or HAp granules, although both materials showed comparable overall bone formation, when implanted in ovariectomized and non-ovariectomized rats^72^. A larger amount of mineralized bone was observed in the center of the defect in HAp group, mainly in ovariectomized rats, whereas at the periphery of the defect the bone area was higher using Sr-doped granules, irrespective of ovariectomy.

Granulation tissue with blood vessels and increased collagen deposition were observed at 60 days post-implantation in Sr-hybrid filled defects. Several studies have correlated the color of birefringent collagen fibers under polarized light with different collagen types^73, 74^. Our results show an increase in thicker red collagen fibers in Sr-hybrid group. These fibers are associated with type I collagen, the main type found in bone tissue. It has been shown that the incorporation of Sr in biomaterials may decrease the number of osteoclasts significantly, but these were nevertheless closely associated with newly formed trabeculae, indicating activated bone remodeling^75^. A quantitative analysis of TRAP positive cells was not performed. However, the presence of osteoclasts in the center of the defect supports the higher bone remodeling found in the Sr-hybrid group. Although the micro-CT calculations did not reveal a statistically different bone volume, these histological findings sustain the evidence of an improved response with the Sr enriched material.

The use of methylmethacrylate embedding histological technique allowed for the study of both bone and material without decalcification. The technique was optimized in our lab^76^ using an exothermic process. One of the disadvantages of the procedure is the inability to perform immunohistochemistry studies due to loss of antigenicity, which is worth exploring in future works.

With the use of Sr releasing systems, cardiovascular safety is of concern. Current guidelines indicate that orally administered Sr ranelate should be avoided in patients with past or present history of ischemic heart disease, peripheral arterial disease and/or cerebrovascular disease or uncontrolled hypertension, due to an observed increase of cardiovascular events^12^. Although previous reports have shown that ionic Sr can be added to calcium phosphates and ceramics, potentially stimulating bone formation locally, the risk of systemic adverse effects has been rarely reported. Baier *et al*. studied the addition of Sr to calcium-phosphate cement in a distal methaphyseal femoral defect in ovariectomized rat model. Results have shown faster osteointegration of the implant with the addition of Sr, and Sr serum concentrations of 10.87 ± 4.16 μg/l were found 1 month post-implantation^35^. The systemic Sr levels were very low when compared to those found upon oral Sr ranelate treatment^77^. In the present study, Sr concentration was assessed both in the serum and organs with excretory/filtration functions, as well as the histology of these organs. Serum Sr concentration in operated animals was found to be similar to non-operated and in the same range as previously reported^35^. No statistically significant difference was observed between Sr-hybrid implanted and empty defects, both in serum and organs. Similarly, Sr concentration levels do not seem to be increased compared to normal levels found in the liver of Wistar rats (~0.2 ug/g of dry weight^78^). These results, together with the absence of morphological changes in histological sections of the organs suggest that Sr release is restricted to the defect site, corroborating the safety of this osteoinductive hybrid system.

## Conclusions

We evaluated the *in vivo* response of an injectable Sr-rich hybrid system composed of Sr-doped HAp microspheres embedded in Sr-crosslinked RGD-alginate hydrogel intended for bone regeneration. Sr-hybrid system led to an increased bone formation in both center and periphery of a critical-sized defect compared to a non Sr–doped similar system, where new bone formation was restricted to the periphery. Besides promoting earlier new bone formation, Sr-hybrid system was also found to stimulate higher cell colonization with increased deposition of thick collagen fibers in the center of the defect. Importantly, our results suggest that only local release of Sr from the material was obtained, since no statistically significant differences on Sr concentration were detected in retrieved organs or serum. Together, these data demonstrate that the incorporation of Sr improved the osteoinductive properties of the hybrid system leading to higher bone regeneration without inducing detrimental side effects currently associated with other Sr-based therapeutic strategies. The Sr-hybrid material stands as a promising approach for bone regeneration strategies through minimally invasive procedures.

## Materials and Methods

### Preparation of the injectable hybrid materials

For the preparation of the hybrid system, RGD-alginate was combined with Sr-doped hydroxyapatite (HAp) or HAp microspheres and crosslinked by internal gelation with Sr or Ca carbonate, respectively (hereafter designated as Sr-hybrid or Ca-hybrid). These formulations and methodologies were adapted and optimized from previous works using Ca-crosslinked alginate hydrogels^47, 48, 63, 79^.

Ultra-pure (UP) LVG Alginate (Pronova FMC Biopolymers, G content ≥60%, MW 131 ± 13 kDa) was functionalized with RGD peptides as previously described^63^, filtered in 0.22 µm Steriflip units (Millipore), lyophilized and stored at −20 °C until further use. Endotoxin levels were measured in RGD-modified and non-modified UP alginate using the Food and Drug Administration (FDA) approved Endosafe™-PTS system (Charles River). The analysis was performed and certified by an external entity (Analytical Services Unit, IBET/ITQB) revealing endotoxin levels below 0.1 EU/mL (EU - unit of measurement for endotoxin activity), respecting the US Department of Health and Human Services guidelines for implantable devices.

Sterile RGD-Alginate was dissolved in 0.9% (w/v) NaCl solution under sterile conditions to yield a 4% (w/v) solution, which was thoroughly mixed with an aqueous suspension of SrCO_3_ (Sigma) or CaCO_3_ (Fluka) at SrCO_3_/COOH or CaCO_3_/COOH molar ratio of 1.6. A fresh solution of glucone delta-lactone (GDL, Sigma) was added to trigger gel formation at a final polymer concentration of 3.5% (w/v) and a carbonate/GDL molar ratio of 0.125.

HAp microspheres were prepared as described elsewhere^42^. Briefly, HAp powder (Plasma Biotal) was dispersed in a 3% (w/v) alginate solution (FMC Biopolymers) with a ceramic-to-polymer solution ratio of 0.25. The paste was extruded dropwise into 0.1 M SrCl_2_ (Merck) or 0.1 M CaCl_2_ (Merck) crosslinking solution, to produce Sr-HAp or HAp microspheres, respectively. Microspheres were allowed to reticulate for 30 min in the crosslinking solution, and were then washed in deionized water, dried and sintered at 1200 °C. Upon sintering, the polymer phase is burned out giving rise to a porous network where Sr or Ca ions are incorporated in the ceramic particles. Microspheres with spherical shape and diameter of 500–560 µm were retrieved by sieving and autoclave sterilized for further use. Sterile microspheres were promptly added to the gelling alginate solution to yield 35% in weight of the total mixture, thoroughly homogenized and placed in a 1 mL syringe (Terumo) ready for extrusion of the material.

### Animal surgical procedure

All animal experiments were conducted following protocols approved by the Ethics Committee of the Portuguese Official Authority on Animal Welfare and Experimentation (DGAV) – reference no. 0420/000/000/2012. We used a critical-sized metaphyseal bone defect model adapted from Le Guehennec *et al*.^80^, as previously described^81^. Three months old Wistar Han male rats (Charles River Laboratories) with weight ranging from 300 to 400 g were used. Two different experimental groups (n = 5 animals/group) were analysed: bone defect filled with Sr-hybrid material and bone defect filled with Ca-hybrid material (control material). Animals with empty defects (n = 5) were used as a critical-sized defect model control. Two different time-points were used, 15 days and 60 days, to evaluate the relationship of inflammation and early bone formation, and new bone formation, respectively. Non-operated animals (n = 2, 60 days) and animals with empty defects were used as control for serum Sr quantification and organ histological analysis. The analgesic buprenorphine (0.05 mg per kg), was administrated subcutaneously, 30 minutes before surgery. The animals were then subjected to volatile anesthesia with isofluorane, in a chamber, according to standard procedures of the animal facility (inducing anesthesia with 900 cc O_2_/min, 5% Isofluorane), confirmed by loss of posture and reflexes. Animals were then moved to a clean surgery area and anesthesia was maintained along all time of surgery with a face mask (300 cc O_2_/min, 2.5% Isofluorane). The right knee of each animal was shaved and skin cleaned and disinfected with 70% ethanol. A lateral incision was performed and both skin and muscles were retracted to expose the articular capsule. After arthrotomy, a cylindrical defect with 3 mm diameter and depth of approximately 4 mm was drilled in the anterolateral wall of the lateral condyle of the femur. The defect was washed with physiological saline solution and either filled with a biomaterial or left empty. All materials were prepared in sterile conditions and injected in the femur’s critical defect using a 1 mL syringe. Skin and muscle were sutured and the animal was placed back in its cage. Animals were observed until regaining consciousness. Post-operative care was carried out for 48 hours, where analgesics were given (Buprenorphine) in the same dose as before surgery, every 12 hours, with a subcutaneous injection. Behavior and wound healing were examined along time.

### Sample collection

Fifteen and sixty days post-surgery animals were sacrificed. Animals were kept under volatile anesthesia (Isofluorane) and blood collection was performed by cardiac puncture. Pentobarbital (Eutasil) was administered for euthanizing animals, and femurs and organs (liver, left and right kidneys and spleen) were retrieved. Blood was centrifuged and serum collected and stored at −80  °C until further use. Femurs were cleaned from surrounding soft tissue and immediately placed in 10% (v/v) formalin neutral solution for 4 days, rinsed in phosphate buffered saline (PBS) solution and dehydrated in serial ethanol solutions (50–70%) for 3 days each. Femurs were maintained in 70% ethanol at 4 °C until further use. Organs were also placed in 10% (v/v) formalin solution for 24 h and further processed for paraffin embedding.

### Radiographic analysis

Lateral X-ray of femurs retrieved from animals sacrificed at 15 days post-surgery were obtained using a radiographic system (Owandy). For the remaining animals, an *in vivo* lateral X-ray was also performed at 30 days post-surgery, to allow for a follow-up of defects and materials.

### Micro-computed tomography (micro-CT) analysis

Bone defects and adjacent areas were analyzed using a high-resolution micro-CT (Skyscan 1072 scanner). Specimens (n = 5, 60 days post-implantation) were scanned in high resolution mode, using a pixel size of 19.13 μm and an integration time of 1.7 ms. The X-ray source was set at 91 keV of energy and 110 μA of current. A 1-mm-thick aluminum filter and a beam hardening correction algorithm were employed to minimize beam-hardening artefacts (SkyScan hardware/software).

For all scanned specimens, representative datasets of 1023 slices were used for morphometric analysis. To quantify new bone formation, a volume of interest (VOI), corresponding to the femoral defect volume, was delineated using CTAn software (Skyscan Ltd), to enable quantitative analysis to be performed. Binary images were created using two different thresholds, 50–255 (corresponding to particles and new bone) and 90–255 (just particles), and the respective TV (Total volume) determined. The difference between both TV corresponds to the volume of new bone formed (Bone volume, BV). Additionally, 3D virtual models were generated using an image processing software (ANT 3D Creator v 2.4, SkyScan). The micro-CT threshold was first calibrated from a backscattered image with primarily determined quantitative histological measurements, which was then applied equally to all samples.

### Histological Analysis

Femurs were dehydrated in 100% ethanol for 3 days, at 4 °C, followed by immersion in xylol for 24 h and further embedding in methylmethacrylate and processed for histological analysis as described elsewhere^76, 82^.

Serial 7 µm coronal slides were retrieved and the intermediate region of the defect (1200–1500 µm) was stained with Hematoxylin and Eosin (H&E), Masson’s Trichrome (MT), Picrosirius Red (PSR) and Tartarate-resistant acid phosphatase (TRAP)-light green (LG) staining. Briefly, for H&E, undeplastified sections were re-hydrated in deionized water and incubated in Gill’s Hematoxylin for 6 min and counterstained with alcoholic Eosin Y for 1 min. For MT staining, an MT kit (Sigma-Aldrich) was used according to manufacturer’s instructions in undeplastified sections. TRAP staining was performed according to manufacturer using a TRAP kit (Sigma) and counterstained with LG 0.1% (v/v) in deplastified section with xylol overnight. Sections were visualized under a light microscope (DP25, Olympus) and imaged. For PSR staining, sections were deplastified, hydrated in decreasing ethanol gradient to de-ionized water, stained for 6 min in Celestine blue and another 6 min in Gill’s Hematoxylin. After a 10 min washing step in water, sections were stained with Sirius Red for 1 h, washed with acidified water, dehydrated and mounted. Sections were imaged through polarization lens under a light microscope (Axiovert 200 M, Zeiss) using MosaiX software.

Regarding the retrieved organs, paraffin sections of 3 μm thickness were sequentially obtained and stained with H&E.

Peripheral bone thickness was determined as the average thickness of twenty different random locations (arrow in Fig. 4Ac” and d”) of bone found around the defect area using MT stained sections. AxioVision software was used for the measurements (n = 5 animals/group). The area of residual material found within the defect was measured by the same user, manually delimiting the area of the hybrid (microspheres and alginate) in MT stained sections. Alginate and microspheres retain staining and have a different texture, allowing for the easy identification of the material. MosaicJ (ImageJ software, n = 5 animals/group, 3 sections/animal) was used for the assembly of microscopic images at 20x magnification and area values were obtained in ImageJ.

Birefrigent green and red fibers were quantified as the percentage of thin/type III and thicker/type I collagen fibers, respectively^73, 74^. The collagen area within the central region of the defect (diameter = 2.4 mm) was quantified in ImageJ software (n = 5 animals/group, 3 images/animal). Sections were stained simultaneously and images acquired in the same day with the same parameters.

Serial 7 µm coronal slides were also analysed by Scanning Electron Microscopy/Energy-dispersive X-ray spectroscopy (SEM/EDS) using a High Resolution (Schottky) Environmental Scanning Electron Microscope with X-Ray Microanalysis and Electron Backscattered Diffraction analysis: Quanta 400 FEG ESEM/EDAX Genesis X4 M. Samples were coated with an Au/Pd thin film, by sputtering, using the SPI Module Sputter Coater equipment.

### Systemic Sr quantification by Inductively Coupled Plasma - Atomic Emission Spectroscopy (ICP-AES)

Sr levels in serum and organs (spleen, liver and kidneys) were quantified by ICP-AES (Horiba Jobin-Yvon, Ultima spectrometer, generator RF of 40,68 MHz). Serum samples (n = 6 samples/group/time point and n = 2 non-operated) were diluted 5 times in 1% Suprapur nitric acid (Fluka) as described elsewhere^83^. Spleen, liver and kidneys were digested in Suprapure nitric acid (n = 4 samples/group/time point). Before use, all glass materials were washed and then immersed in a 20% (v/v) nitric acid solution for at least 1 day in order to eliminate possible contaminations with Sr or other impurities from the vessels walls. Organs (~300 mg) were dried in a microwave (MARS-X 1500 W, CEM) configured with a 14 position carousel. An aliquot of 10 mL of Suprapur nitric acid was added and microwave digestion proceeded during 55 min, according to microwave digestion program (Supplementary - Table 1). The solutions were concentrated until 1 mL and preserved at −20 °C until Sr determination. The limit of detection (LOD – 1 μg/L) and limit of quantification (LOQ – 5 μg/L) for Sr were adequate for the expected concentration range of the samples.

### Statistical Analysis

Statistical analysis was performed using non-parametric Mann–Whitney test with GraphPad Prism Program. A value of p < 0.05 was considered statistically significant: *p < 0.05; **p < 0.01; ***p < 0.001.

## Electronic supplementary material

Microwave digestion program
