# Supplementary material for: Injectable hybrid system for strontium local delivery promotes bone regeneration in a rat critical-sized defect model
Source: Sci Rep. 2017 Jul 11;7:5098. doi: 10.1038/s41598-017-04866-4 (PMC5506032; doi:10.1038/s41598-017-04866-4)
Supplement: Supplementary file 1 — Microwave digestion program [file 41598_2017_4866_MOESM1_ESM.pdf]

Injectable hybrid system for strontium local delivery promotes bone regeneration in a rat critical-sized defect model

Ana Henriques Lourenço[1,2,3 *], Nuno Neves[1,2,4 *], Cláudia Ribeiro-Machado [1,2], Susana R. Sousa[1,2,5], Meriem Lamghari[1,2], Cristina C. Barrias[1,2], Abel Trigo Cabral[4], Mário A. Barbosa[1,2,6] and Cristina C. Ribeiro[1,2,5 #]

[1]i3S - Instituto de Investigação e Inovação em Saúde, Universidade do Porto, Rua Alfredo Allen, 208, 4200 - 135 Porto, Portugal

[2]INEB - Instituto de Engenharia Biomédica, Universidade do Porto, Rua Alfredo Allen, 208, 4200 - 135 Porto, Portugal

[3]Faculdade de Engenharia, Universidade do Porto, Rua Dr. Roberto Frias, s/n, 4200-465 Porto, Portugal

[4]Faculdade de Medicina, Universidade do Porto, Serviço de Ortopedia, Alameda Prof. Hernâni Monteiro, 4200-319 Porto, Portugal

[5]ISEP – Instituto Superior de Engenharia do Porto, Instituto Politécnico do Porto, Rua Dr. António Bernardino de Almeida 431, 4249-015, Porto, Portugal

[6]ICBAS - Instituto de Ciências Biomédicas de Abel Salazar, Universidade do Porto, Rua de Jorge Viterbo Ferreira n. 228, 4050-313 Porto, Portugal

[*] *These authors contributed equally to this work.*

[#] Corresponding author: Cristina C. Ribeiro (cribeiro@ineb.up.pt)

Address: i3S – Instituto de Investigação e Inovação em Saúde, Universidade do Porto

Rua Alfredo Allen, 208

4200 – 135 Porto

Phone: +351 220 408 800

Supplementary Data

Table 1 - Microwave digestion program.

Stages	1	2	3
Power (W)	600	600	600
Time (min.)	5	10	10
Temperature (control, °C)	50	100	175
Hold (min.)	10	10	15
